# Isolation of virulent phages against multidrug-resistant *Acinetobacter baumannii* recovered from inanimate objects of Jimma Medical Center, Southwest Ethiopia

**DOI:** 10.1186/s12879-023-08823-7

**Published:** 2023-11-22

**Authors:** Terefe Hailemichael, Lencho Girma, Paulos Fissiha, Alene Geteneh, Tesfaye Kassa

**Affiliations:** 1 Department of Medical Laboratory Science, Mizan Aman College of Health Sciences, Aman, Ethiopia; 2 Department of Medical Laboratory Science, College of Health Sciences, Bonga University, Bonga, Ethiopia; 3 Amhara Public Health Institute (APHI), Bahir Dar, Ethiopia; 4 Department of Medical Laboratory Science, College of Health Sciences, Woldia University, Woldia, Ethiopia; 5 School of Medical Laboratory Science, Jimma University, Jimma, Ethiopia

**Keywords:** MDR *A. Baumannii*, Biofilm, Phage, Inanimate objects, Jimma Medical Center

## Abstract

**Background:**

Because of the multidrug resistance features of *Acinetobacter baumannii*, endurance to diverse conditions, and causing health fatalities in healthcare settings, the global health system is looking for the development of new antimicrobials for such bacteria. As the new antimicrobial drugs pipeline is running dry, it is imperative to look for eco-friendly bio-control strategies. In this regard, phages are one to combat the biofilm producer and MDR *A. baumannii*. Thus, the study aimed to isolate and examine the role of phages against biofilm producers and MDR *A. baumannii* from inanimate objects at Jimma Medical Center (JMC), Ethiopia.

**Method:**

Institution-based cross-sectional study was conducted from June to November 2019. A total of 309 swab samples were collected from inanimate objects and the environment in JMC. Isolation of *A. baumannii*, antimicrobial susceptibility testing, and biofilm detection were carried out according to standard protocol. Kirby Bauer disk diffusion and microliter plate were methods for AST and biofilm detection, respectively. Specific phage was isolated and characterized from sewage at JMC compound. The data were analyzed by SPSS version 25.0, and chi-square (X^2^) cross-tabulation was used to determine the correlation of variables. A *P*-value of < 0.05 was considered a statistically significant association.

**Result:**

*A. baumannii* from inanimate objects and surfaces of different environments at JMC was detected in 6.5% of the samples. From 20 of the isolates, 85% were biofilm producers, and 60% were MDR. The lytic phage isolated specifically against *A. baumannii* was found host specific, and thermally stable ranging from 10–50°C. The phage was active against 42% of MDR *A. baumannii*, 40% of both biofilm-producing and MDR *A. baumannii* (MDRAB), and 35.3% of the biofilm-producing isolates.

**Conclusion:**

The good activity of phages towards MDRAB isolates, its biofilm degradation capability, thermal stability, and host specificity in our study encourages viewing the potential use of phages as a bio-control agent besides the routine cleansing agents. Therefore, we recommend isolation of specific phages in the eradication of MDRAB from health facilities with additional efforts to characterize in detail and assess their efficacy in animal models.

**Supplementary Information:**

The online version contains supplementary material available at 10.1186/s12879-023-08823-7.

## Background

The emergence of multidrug-resistant (MDR) bacterial species is a hot global public health challenge. Among these challenging MDR bacteria, WHO has identified six opportunistic pathogens known as ESKAPE (*Enterococcus faecium*, *Staphylococcus aureus*, *Klebsiella pneumoniae*, *Acinetobacter baumannii*, *Pseudomonas aeruginosa*, and *Enterobacter species*) for research and development of new antibiotics [1]. The abbreviation ESKAPE indicates the bacteria`s ability to “escape” the killing of antibiotics and resist eradication by conventional therapies as well, leading to extensive morbidity and mortality among admitted patients within healthcare settings [2].

*A. baumannii* is a non-fermentative, non-motile, non-fastidious, catalase-positive, oxidase-negative, aerobic Gram-negative coccobacilli opportunistic pathogen responsible for different infections including pneumonia, bloodstream, wound, and urinary tract infections [3], and meningitis among patients in intensive care units [4]. It is also known as the “Iraqibacter” because of its emergence during the Iraq and Afghanistan war among US soldiers causing severe infections [5]; appears as a real challenging superbug for clinicians [6]. Because of its MDR features, the high mortality rate (up to 23% for hospital-admitted patients and up to 43% among ICU patients [7]), endurance on inanimate objects [8], biofilm-assisted survival in harsh environments [9, 10]; *A. baumannii* has got due attention globally.

Despite the efforts of the scientific community to develop new effective medications against the MDR pathogen, the number of antibiotics joining the market is running dry, and humanity is threatened [11]. So far, 300 million people are expected to die globally for the next 30 years, and a 60 to 100 trillion USD loss is expected if antimicrobial resistance (AMR) continues to be uncontrolled [12]. The strike due to AMR pathogens would be worse, particularly in poor nations like Ethiopia. Recent literature redirected the focus to an emerging eco-friendly, bio-control strategy as an alternative to/with antibiotics against resistant bacteria [13]. Among the options, bacteriophages, or phages in short, are emerging as new specific and rapid killing machines against MDR pathogens [14, 15]. Sharing the concern, this study aimed to investigate the role of phages against MDR *A. baumannii* (MDRAB) and biofilm-producing isolates recovered from inanimate objects at JMC, Ethiopia.

## Materials and methods

### Study setting

A health facility-based cross-sectional study was conducted from June to November 2019 in JMC where the facility is found in Jimma town, southwest of Ethiopia. Recently JMC has been providing different medical specialty services and has a bed capacity of 650 for over 15 million populations in the catchment area [16].

### Data collection and processing

The number of rooms in the health facility was sampled based on the CDC, 2010 guideline for evaluating environmental cleaning; where sampling 15% of the rooms is considered reasonably representative for hospitals with ≥ 150 beds [17]. A total of 309 swab samples were collected randomly from high-touch surfaces in 37 rooms in five wards (13 surgical, 8 pediatrics, 7 medical, 6 gynecology and obstetrics rooms and 3 ICU rooms (surgical ICU, medical ICU, and pediatrics ICU)) in the morning between 8:30 − 9:00 A.M after routine morning cleaning. The number of swabs collected from each of the wards’ rooms i.e. surgical, pediatrics, medical, gynecology and obstetrics, and ICU wards were 99, 66, 58, 47, and 39, respectively. Each sampled swab was properly homogenized in 1mL sterile normal saline. One hundred μL of the sample was aseptically inoculated onto MacConkey agar (Oxoid, Ltd, Hampshire, England) and incubated aerobically at 37 °C for 24 h [18]. The mean colony forming unit per square centimeter (CFU/cm^2^) area was calculated and compared with the standard for high-touch surfaces which is ≤ 5CFU/cm^2^ [19]. Identification of bacteria was done using different characteristics including colony morphology, Gram stain, and biochemical profiling such as catalase, oxidase, citrate utilization test, Kligler iron agar (KIA), Sulfide Indole Motility test, oxidation-fermentation test, growth at 44^0^ C, and inoculated on blood agar to check for hemolysis [20, 21]. *A. baumannii* produces colorless non-lactose fermenting shiny mucoid colonies on MacConkey agar and is the only group member that is capable to grow at 44ºC from the genus [22–24].

### Antimicrobial susceptibility test and biofilm detection

Three to five pure colonies of *A. baumannii* isolates from overnight grown culture were suspended in sterile normal saline. The turbidity of the suspension was checked against 0.5 McFarland standard. Antimicrobial susceptibility testing (AST) was performed using the Kirby Bauer disk diffusion technique on Muller Hinton Agar (Oxoid, Ltd, Hampshire, England). The following antimicrobials were tested: ceftriaxone, 30 *μ*g; ciprofloxacin, 5 *μ*g; gentamicin, 10 *μ*g; ceftazidime, 30 μg; cefepime, 30 μg; imipenem, 10 μg; meropenem, 10 μg; amikacin, 30 μg; doxycyciline, 30 μg; and trimethoprim/sulfamethoxazole, 1.25/23.75 μg) (Liofilchem srl, Italy). Antibiotic discs were placed firmly and incubated at 37ºC for 24 h. The zone of inhibition was measured and interpreted according to the CLSI 2018 recommendations [25]. Reference strains including *P. aeruginosa* ATCC 27853, *K. pneumoniae* ATCC 700603, *S. aureus* ATCC 25923, and *E. coli* ATCC 25922 were used for antimicrobial susceptibility and phage host range testing.

Microtiter plate assay (96 wells) was used to determine biofilm production following the protocol used by Sanchez et al. [26]. The bacterial suspension was added to freshly prepared Trypticase soya broth (TSB) (Oxoid, Ltd, Hampshire, UK) supplemented with 1% glucose and diluted to 0.5 McFarland turbidity standard. Then, 200 μL was added in each microtiter well for each isolate in triplicate and incubated at 37ºC for 48 h. Following incubation, the content of each well was aspirated and washed 3 times gently with sterile phosphate buffer saline (PH 7.2) to remove planktonic bacterial cells. The attached bacteria were fixed with 200 μL of methanol in each well. Then, 250 μl of 0.1% crystal violet solution was added to each well and was kept for 10 min at room temperature. Each microtiter well was washed with PBS saline to remove the staining solution. After plates were allowed to air-dry, 250 μl of 95% ethanol was added to solubilize the crystal violet dye by incubating for 15 min at room temperature. The solubilized content of each well was aspirated and transferred into a new microtiter plate well. The optical density (OD) of each well was measured at 595 nm by using an automatic ELISA Reader (Elisys Uno, Human Germany). The measured optical density (OD) from the triplicate wells was then averaged and the standard deviation was calculated. The cut-off optical density (ODc) was calculated and defined as three standard deviations above the mean OD of the negative control (Trypticase soya broth without bacteria). Based on the average OD produced by bacterial films at a wavelength of 595 nm; *A. baumannii* isolates were classified as bacterial OD < ODc = biofilm non-producer; OD > ODc, but < 2 ODc = weak biofilm producer; OD > 2 ODc but < 4 ODc = moderate biofilm producer and > 4 ODc = strong biofilm producer as described by Stepanovic and his team [27].

### Bacteriophage isolation, enrichment, and characterization

Bacteriophage specific against *A. baumannii* was isolated from sewage samples collected at four different collection sites in JMC following the standard phage isolation protocol stated by Clokie et al., [28]. 50 ml of each sample was centrifuged at 10,000 rpm for 10 min to remove particulate materials. The supernatants were filtered by a 0.45-micrometer membrane filter. Then 20 ml of filtrate was mixed with an equal volume of double-strength broth containing 5mM MgSo4 along with 2 ml of log phase growth of *A. baumannii* and incubated at 37^0^ C by shaking every 2–4 h. After 24 h incubation the content of the flask was centrifuged at 10,000 rpm at 4^0^ C for 15 min. The supernatant containing phage was passed through a 0.45-micrometer pore membrane filter under aseptic conditions and the filtrate was used for amplification of phage. The spot assay was used to check for the phage activity against A. baumannii [29, 30]. The host bacterial cell suspension (0.1ml) was added to sterile soft agar (0.8%) maintained in a molten state at 45 °C in a water bath and quickly mixed. Then, the mixture was poured into previously prepared nutrient agar plates and two drops (10 μl) of the amplified filtrate were spotted on the plate at two different places. The plates were examined the next day for clearance at the spotted area after incubation at 37 °C for 24 h. Phage activity was examined against known control strains of *P. aeruginosa* ATCC 27853, *K. pneumoniae* ATCC 700603, *S. aureus* ATCC 25923, and *E. coli* ATCC 25922. The temperature stability of bacteriophages was evaluated by incubating the phage suspension at 10, 25, 37, 44, 50, 60, and 65°C for 1 h before overnight incubation with *A. baumannii* to determine if the phage retains its lytic activity against the host bacteria using spot assay [31]. Similarly, the phage-biofilm degradation was assessed [32] along with ciprofloxacin (30 μg/ml) to compare anti-biofilm activity [33], and normal saline was used as a control [27]. To evaluate the biofilm eradication activity of phage, 100 μl of *A. baumannii* culture in the log phase was inoculated into 200ml of Brain heart infusion (BHI) broth. The inoculated broth was aseptically poured into a tip box containing cover glass leaving liquid air interphase for growth of biofilm and incubated for 36 h. After the growth, the cover glass was aseptically removed and washed with sterile phosphate buffer saline (pH 7.2). Then, the biofilm developed on cover glass was treated with bacteriophage or ciprofloxacin (30 μg/ml) or normal saline and incubated for 3 and 36 h [32]. After the respective treatment, biofilm grown on a cover glass was washed gently with sterile phosphate buffer saline (pH 7.2) and stained with (0.1%) crystal violet for 10 min. The stained biofilm was rinsed with sterile distilled water allowed to air dry and put on a clean microscope slide for microscopic examination. The cover glass treated with normal saline was used as a control [27, 33].

### Statistical analysis

Data were checked and cleared for completeness and exported to SPSS for analysis. The chi-square (χ2) test was used to determine the association between variables. A *P*-value of < 0.05 was considered statistically significant for association.

## Results

**Isolation and enumeration of*A. baumannii***.

From a total of 309 health facility high-touch surfaces bacteriological samples, 184 (59.5%) showed Gram-negative bacterial growth on MacConkey agar plates. However, the recovery rate of *A. baumannii* was 6.5% (n = 20) or about 11% from among Gram-negative. The distribution of *A. baumannii* from the inanimate objects was seven from the floor, four from tables, three from bed frame, two from the door handle, and one each from the oxygen control valve, wash sink, ventilator screen, and circuit of mechanical ventilation. The number of *A. baumannii* recovered varies significantly with inanimate objects (*P* < 0.005), but not with wards (Table 1).

**Table 1:** Distribution of *A. baumannii* on inanimate objects at JMC, June-November, 2019

Sampled objects	Growth on MacA.^#^		Presence of *A. baumannii*			*P*-value
	Yes	No	Yes	No	Total	
Table	30	7	4(10.8%)	33 (89.1%)	37	0.033
Bedframe	17	20	3 (8.1%)	34 (91.89%)	37	0.114
Door handle	22	15	2 (5.4%)	35 (94.5%)	37	0.362
Floor	36	1	7 (18.9%)	30 (81%)	37	0.001
Others*	79	82	4 (2.5%)	157 (97.5%)	161	
Total	184	125	20 (6.5%)	289 (93.5%)	309	

*Others include a wall bulb switch, hand wash sink, IV stand, oxygen control valve, locker handle, circuits of mechanical ventilation, ventilator screen, ventilator screen button, and pulse oximetry

# MacConkey Agar medium

Evaluation of the environment for cleaning and disinfection process of frequently hand contact surfaces indicated the possibility of an increased risk of infection for patients from the environment whatever the type of organism is. The colony count in all the rooms was above standard for high touch surface ≤ 5CFU/cm^2^.

### Antibiotic resistance profile and biofilm production of ***A. baumannii***

The antimicrobial resistance patterns of *A. baumannii* demonstrated an increased level of resistance to imipenem (100%), followed by ceftriaxone and ceftazidime (95%), Cefepime (80%), Cotrimoxazole (70%), and Meropenem (60%). However, they were sensitive to Doxycycline (100%) followed by Amikacin (95%) (Table 2). Of the 20 isolates recovered, 85% (n = 17) of them were biofilm producers, and recovery of biofilm-forming and MDR *A. baumannii* was shown to vary with admission wards (Table 3).

**Table 2:** Antibiotic resistance profiling of *A. baumannii* isolates at JMC, June-November, 2019

Antibiotic tested	Drug susceptibility (n = 20)		
	Susceptible, No (%)	Intermediate, No (%)	Resistant, No (%)
Gentamicin	15(75)	2(10)	3(15)
Ciprofloxacin	15(15)	0	5(25)
Cefepime	2(10)	2(10)	16(80)
Ceftazidime	1(5)	0	19(95)
Ceftriaxone	1(5)	0	19(95)
Meropenem	6(30)	2(10)	12(60)
Imipenem	0	0	20(100)
Cotrimoxazole	6(30)	0	14(70)
Amikacin	18(90)	1(5)	1(5)
Doxycycline	20(100)	0	0

**Table 3:** Biofilm production and level of MDR profiling of *A. baumannii* isolates at JMC, June-November, 2019

No. of A. *baumannii* isolated per wards	MDR		Biofilm production	
	Yes	No	Yes	No
Surgical ward (n = 5)	2	3	3	2
Medical ward (n = 4)	3	1	4	0
Pediatrics ward (n = 4)	1	3	3	1
Gyn & Obs ward (n = 2)	1	1	2	0
ICU (n = 5)	5	0	5	0
**Total (n = 20, 100%)**	**12(60%)**	**8(40%)**	**17(85%)**	**3(15%)**

60% (12/20) of the isolates were MDR *A. baumannii*. Similarly, nearly 59% of (10/17) the biofilm producer *A. baumannii* isolates were MDR. Although the association is indeterminate, being MDR isolate was shown to vary with inanimate objects too; where 5 out of the 12 MDRAB isolates were recovered from the facility floor. The isolates’ OD value was cross-tabulated against the number of antibiotics resisted (Supplementary Table 1). The Spearman correlation of OD values of biofilm assay had shown a significant association with the number of *A. baumannii* isolates resistant to antibiotics (r = 0.635, *p*-value = 0.027).

### Activity of phages against biofilm-forming and MDR ***A. baumannii*** isolates

**Fig. 1:**
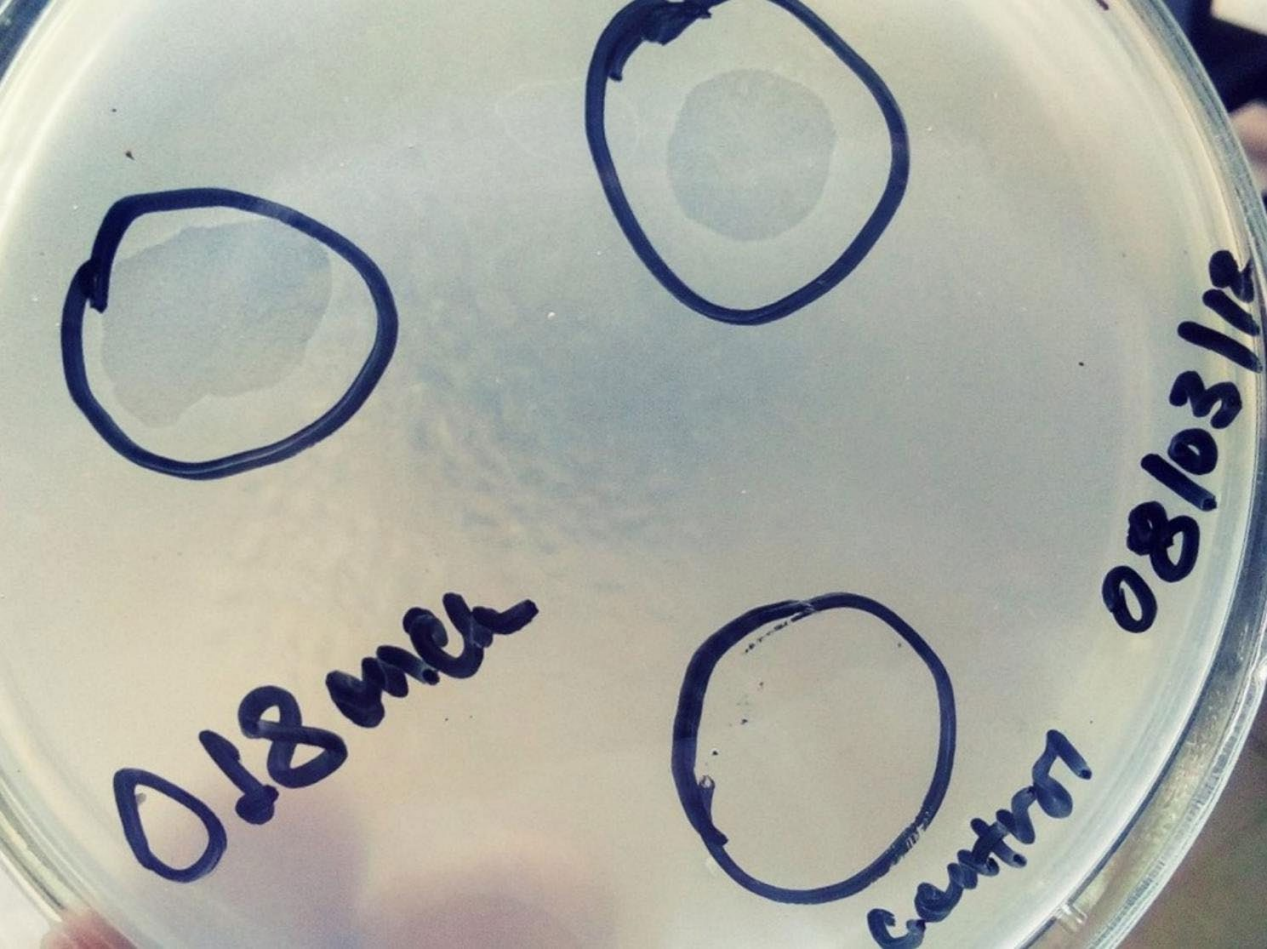
Spot assay showing complete clearance of spotted area which indicates lytic phage activity

Of the four sewage samples processed, one lytic phage specific against MDR *A. baumannii* was isolated. Seven of the 20 *A. baumannii* isolates (biofilm producer, MDR, or both biofilm producer and MDR) tested have shown lysis by the phage fully or partially (Table 4). In this study, 40% of both biofilm producers and MDRAB were potentially affected by the virulent lytic pages. The phage has specific lytic activity against *A. baumannii* isolates (Fig. 1) but non-lytic against other control reference bacterial strains including *P. aeruginosa* ATCC 27853, *K. pneumoniae* ATCC 700603, *S. aureus* ATCC 25922, and *E. coli* ATCC 25922.

**Table 4:** Lytic activity of phage against MDR and/or biofilm producer *A. baumannii* isolates at JMC June-November, 2019

Isolate name	MDR status	Biofilm production	Phage lytic activity
AB1	MDR	Biofilm producer	No lyses
AB2	MDR	Biofilm non-producer	No lyses
AB3	Non-MDR	Biofilm non-producer	No lyses
AB4	Non-MDR	Biofilm producer	No lyses
AB5	Non-MDR	Biofilm producer	No lyses
AB6	Non-MDR	Biofilm producer	Lyses
AB7	Non-MDR	Biofilm producer	Partial lyses
AB8	MDR	Biofilm non-producer	Lyses
AB9	Non-MDR	Biofilm producer	No lyses
AB10	MDR	Biofilm producer	No lyses
AB11	MDR	Biofilm producer	Lyses
AB12	MDR	Biofilm producer	Lyses
AB13	MDR	Biofilm producer	No lyses
AB14	MDR	Biofilm producer	Lyses
AB15	MDR	Biofilm producer	Lyses
AB16	Non-MDR	Biofilm producer	No lyses
AB17	MDR	Biofilm producer	No lyses
AB18	MDR	Biofilm producer	No lyses
AB19	Non-MDR	Biofilm producer	No lyses
AB20	MDR	Biofilm producer	No lyses

**Fig. 2:**
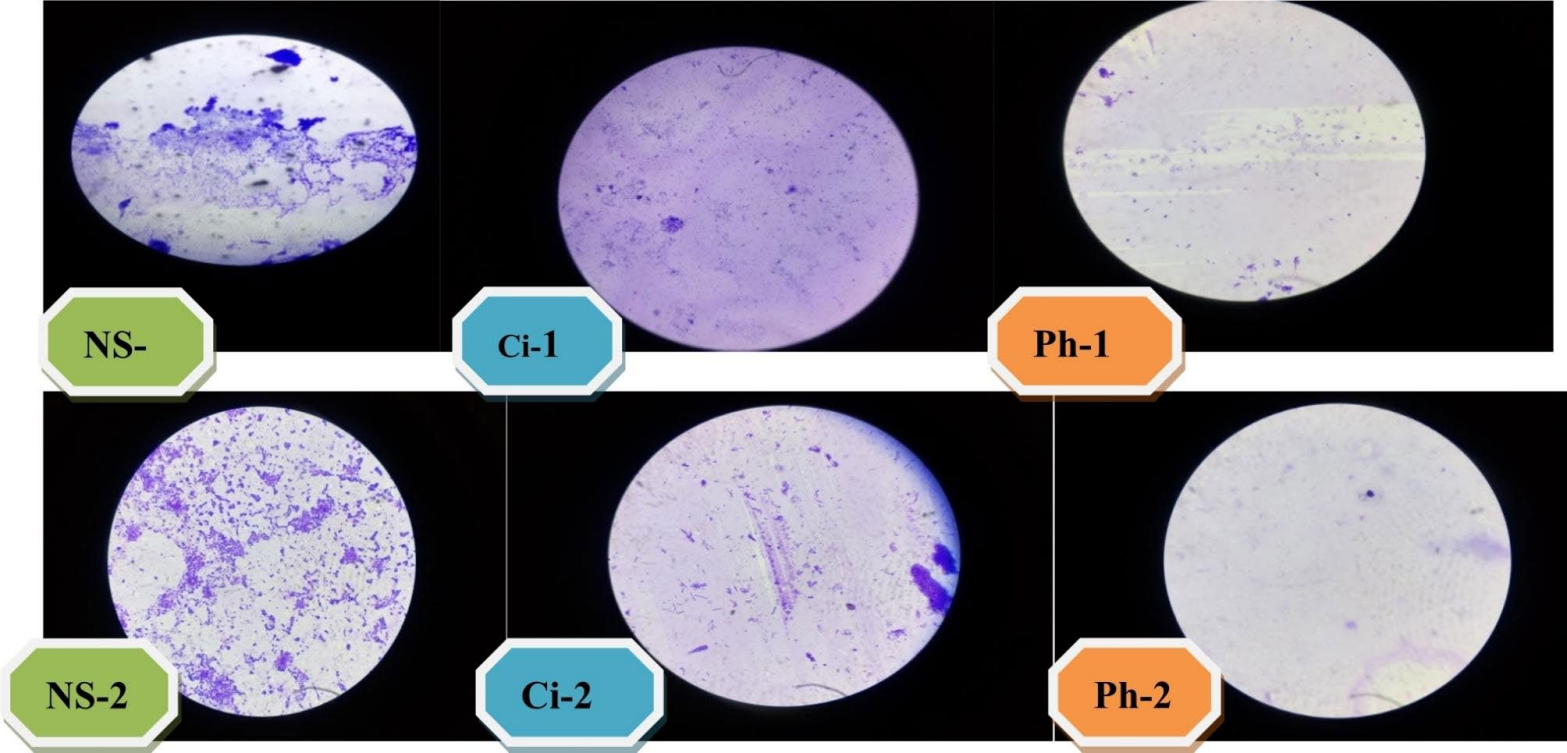
Illustrating biofilm eradication by phage isolates compared to ciprofloxacin and normal saline N.B: NS-1, Ci-1 and Ph-1 cover glass were treated with normal saline, ciprofloxacin, and phage respectively for 3 h whereas NS-2, Ci-2 and ph-2 cover glass were treated with normal saline, ciprofloxacin, and phage for 36 h, respectively Biofilm containg cover glass treated in phage and ciprofloxacillin showed variable eradiction in different time frame. The phage treated showed more eradication. The normal saline treated cover glass showed growth of the bacteria

Temperature stability of the phage at 10^6^ PFU/ml was tested by incubating the phage suspension at 10, 25, 37, 44, 50, 60, or 65°C for 1 h before overnight incubation of phages with the host bacteria. Thus, the phage was active in lysing the host bacteria at 10 to 50 °C but did not at 60°C and beyond. The phage biofilm degradation was examined relative to ciprofloxacin (30 μg/ml) and normal saline as control. Accordingly, phages were effective in eradicating biofilm producers and MDRAB isolates more efficiently than ciprofloxacin (30 μg/ml) as evaluated microscopically in this study (Fig. 2).

## Discussion

The detection rate of *A. baumannii* in JMC facility environment including the surface of the floor, table handle, bed frames, and other frequent hand touch inanimate objects was 6.5% with its maximal recovery from ICU surfaces. The finding is numerically comparable with studies reported from Algeria (7.7%) [34], Brazil (9.5%) [35], and France (4.9%) [36]. But, it was quite lower relative to findings from Iran (17%) [37] and Jordan (49.7%) [38]. This epidemiologic variation could be attributed by the difference in health facilities, the neatness of settings, and adherence to infection prevention and control implementation strategies. Though the medical center rooms were disinfected with a 1:10 concentration of 5% bleach three times a day, *A. baumannii* was isolated from inanimate surface samples. This is an indication of a potential outbreak of *A. baumannii* infections as all the sampled wards were contaminated [34, 35, 37].

The feature of *A. baumannii* being MDR and its capability to remain viable in soil, and environmental contamination of health facilities leads to a global public health challenge [39]. Briefly in this study, 60% of the isolates were MDR (and 85% biofilm producers). Higher prevalence of the bacterium in health settings has been known in different studies such as in Brazil (98.8%) [35], and China (65% and more) [3] whereas less prevalently in Maryland, USA (9.8%) [40]. This could be due to its high adaptability of harsh environmental conditions [4, 9, 10, 39]. As a result, different scientific reports suggest the use of alternative antimicrobial agents and in this regard, the application of phages was endorsed as a newly emerged potential therapeutic option against MDR pathogens [14, 41–44]. With these underlying reasons, the current work investigated the use of specific phage against MDR, and biofilm producer *A. baumannii* isolates recovered from the hospital inanimate objects.

In this pilot study, the phage isolated against one of the MDR bacterium, *A*. *baumannii* had shown full or partial lytic activities against 35 to 42% of biofilm producers, MDRAB or both biofilm producers and MDRAB isolates, (Table 4). The phage isolated was able to lyse only *A. baumannii* isolates in contrast to other ATCC reference strains. The thermal stability ranged from 10 to 50 °C for 1 h [45] with possible variation with the host bacterial strain [46]. This finding is supported by previous studies [47], and even with different bacterial species [32, 41] and animal models used [42, 44, 46]. In addition, phages were more active at deterring bacterial resistance (42%) than degrading biofilms (35.3%) as demonstrated in the previous study [41]. As a limitation, our study merely depends on the phenotypic characterization of phages, and phage-biofilm clearance. In line with other literatures, this study can decipher the most abundant biological entities that have the potential to be used on inanimate objects and environments in health facilities as a biological control as well as a therapeutic candidate against multidrug-resistant and biofilm-producing *A. baumannii* isolates.

## Conclusion

The detection of substantial MDRAB isolates in inanimate objects and environments of the medical center is an indication of the potential occurrence of MDRAB-associated outbreaks in the study setting unless proper decontamination strategies are in place. The good sensitivity of MDRAB isolates, biofilm degradation, thermal stability, and host specificity of phages in our study aspired to potentially identify them as a biocontrol or decontaminating agent from sewage sources in Jimma Medical Center besides the routine cleansing agents. Therefore, we recommend further efforts to characterize phages against emerging MDRAB isolates in detail.

### Electronic supplementary material

Below is the link to the electronic supplementary material.

Supplementary Material 1

## Data Availability

The datasets used or analyzed in the present study are available with the corresponding author upon reasonable request.

## References

[1] Mulani MS, Kamble EE, Kumkar SN (2019). Emerging strategies to Combat ESKAPE pathogens in the era of Antimicrobial Resistance: a review. Front Microbiol.

[2] Ma YX, Wang CY, Li YY (2020). Considerations and caveats in combating ESKAPE pathogens against nosocomial Infections. Adv Sci.

[3] Gao L, Lyu Y, Li Y (2017). Trends in Drug Resistance of Acinetobacter baumannii over a 10-year period: Nationwide Data from the China Surveillance of Antimicrobial Resistance Program. Chin Med J (Engl).

[4] Espinal P, Marti S, Vila J (2012). Effect of biofilm formation on the survival of Acinetobacter baumannii on dry surfaces. J Hosp Infect.

[5] Eze EC, Chenia HY, El Zowalaty ME (2018). Acinetobacter baumannii biofilms: effects of physicochemical factors, virulence, antibiotic resistance determinants, gene regulation, and future antimicrobial treatments. Infect Drug Resist.

[6] Vázquez-López R, Solano-Gálvez SG, Juárez Vignon-Whaley JJ (2020). Acinetobacter baumannii Resistance: a real challenge for clinicians. Antibiotics.

[7] Kazemi H, Yadegarynia D, Rahmati Roodsari S et al. Evaluation of Antimicrobial susceptibility among Acintobacter Baumannii by E-Test Method at Khatam-Al-Anbia Hospital during 2013–2015. Zahedan J Res Med Sci. 2017;In Press(In Press).

[8] Nobrega FL, Costa AR, Kluskens LD (2015). Revisiting phage therapy: new applications for old resources. Trends Microbiol.

[9] Pour NK, Dusane DH, Dhakephalkar PK (2011). Biofilm formation by Acinetobacter baumannii strains isolated from urinary tract Infection and urinary catheters. FEMS Immunol Med Microbiol.

[10] Qi L, Li H, Zhang C (2016). Relationship between Antibiotic Resistance, Biofilm formation, and Biofilm-Specific Resistance in Acinetobacter baumannii. Front Microbiol.

[11] Oduor J, Kadija E, Kiljunen S (2020). What is next after antibiotics?. Int J Infect Dis.

[12] O’Neill J. ‘Review on Antimicrobial Resistance.Antimicrobial Resistance: Tackling a crisis for the health and wealth of nations. 2014.

[13] Hauser AR, Mecsas J, Moir DT (2016). Beyond antibiotics: New Therapeutic approaches for bacterial Infections. Clin Infect Diseases: Official Publication Infect Dis Soc Am.

[14] Popova AV, Zhilenkov EL, Myakinina VP (2012). Isolation and characterization of wide host range lytic bacteriophage AP22 infecting Acinetobacter baumannii. FEMS Microbiol Lett.

[15] D’Accolti M, Soffritti I, Piffanelli M (2018). Efficient removal of hospital pathogens from hard surfaces by a combined use of bacteriophages and probiotics: potential as sanitizing agents. Infect drug Resist.

[16] Pritsch M, Zeynudin A, Messerer M (2017). First report on bla (NDM-1)-producing Acinetobacter baumannii in three clinical isolates from Ethiopia. BMC Infect Dis.

[17] Alice Guh P, Carling, Workgroup EE. Options for Evaluating Environmental Cleaning. 2010.

[18] Monica Cheesbrough. District laboratory practice in tropical countries volume II: microbiology. Cambridge (UK): 2006.

[19] Dancer SJ (2004). How do we assess hospital cleaning? A proposal for microbiological standards for surface hygiene in hospitals. J Hosp Infect.

[20] Dias C, Borges A, Oliveira D (2018). Biofilms and antibiotic susceptibility of multidrug-resistant bacteria from wild animals. PeerJ.

[21] Ozorkwo CAN, Chekwube L (2017). High antibiotics resistance observed inAcinetobacter Baumannii isolated from South East Nigeria. EC Microbiol.

[22] Tripathi PC, Gajbhiye SR, Agrawal GN (2014). Clinical and antimicrobial profile of Acinetobacter spp.: an emerging nosocomial superbug. Adv Biomed Res.

[23] Asif M, Alvi IA, Rehman SU (2018). Insight into Acinetobacter baumannii: pathogenesis, global resistance, mechanisms of resistance, treatment options, and alternative modalities. Infect Drug Resist.

[24] Babapour E, Haddadi A, Mirnejad R (2016). Biofilm formation in clinical isolates of nosocomial Acinetobacter baumannii and its relationship with multidrug resistance. Asian Pac J Trop Biomed.

[25] CLSI (2018). Performance standards for Antimicrobial susceptibility testing.

[26] Carlos JS Jr, Mende K, Beckius ML et al. Biofilm formation by clinical isolates and the implications in chronic Infections. BMC Infect Dis. 2013;13(47). 10.1186/1471-2334-13-47 PMC3568419 23356488

[27] Stepanovic S, Vukovic D, Hola V (2007). Quantification of biofilm in microtiter plates: overview of testing conditions and practical recommendations for assessment of biofilm production by staphylococci. APMIS.

[28] Clokie MR, Kropinski AM, Lavigne R. Bacteriophages: Springer; 2009.

[29] Cerveny KE, DePaola A, Duckworth DH (2002). Phage therapy of local and systemic Disease caused by Vibrio vulnificus in iron-dextran-treated mice. Infect Immun.

[30] Kumari S, Harjai K, Chhibber S (2010). Isolation and characterization of Klebsiella pneumoniae specific bacteriophages from sewage samples. Folia Microbiol (Praha).

[31] Verma V, Harjai K, Chhibber S (2009). Characterization of a T7-like lytic bacteriophage of Klebsiella pneumoniae B5055: a potential therapeutic agent. Curr Microbiol.

[32] Israel Gudina Z, Gizachew D, Woyessa (2018). Isolation of Bacteriophage and Assessment of its activity against Biofilms of Uropathogenic Escherichia coli in Jimma Town, South Western Ethiopia. Am J Curr Microbiol.

[33] Manohar P, Loh B, Nachimuthu R et al. Phage-antibiotic combinations to control Pseudomonas aeruginosa-Candida two-species biofilms. bioRxiv. 2022. 10.1038/s41598-024-59444-2 PMC11039464 38653744

[34] Zenati K, Touati A, Bakour S (2016). Characterization of NDM-1- and OXA-23-producing Acinetobacter baumannii isolates from inanimate surfaces in a hospital environment in Algeria. J Hosp Infect.

[35] Raro OHF, Gallo SW, Ferreira CAS (2017). Carbapenem-resistant Acinetobacter baumannii contamination in an intensive care unit. Rev Soc Bras Med Trop.

[36] Kuczewski E, Henaff L, Regard A et al. Bacterial cross-transmission between Inanimate surfaces and patients in Intensive Care units under real-world conditions: a repeated cross-sectional study. Int J Environ Res Public Health. 2022;19(15). 10.3390/ijerph19159401 PMC9367990 35954765

[37] Shamsizadeh Z, Nikaeen M, Nasr Esfahani B (2017). Detection of antibiotic resistant Acinetobacter baumannii in various hospital environments: potential sources for transmission of Acinetobacter Infections. Environ Health Prev Med.

[38] Obeidat N, Jawdat F, Al-Bakri AG (2014). Major biologic characteristics of Acinetobacter baumannii isolates from hospital environmental and patients’ respiratory tract sources. Am J Infect Control.

[39] Gallego L (2016). Acinetobacter baumannii: factors involved in its high adaptability to adverse environmental conditions. J Microbiol Exp.

[40] Thom KA, Johnson JK, Lee MS (2011). Environmental contamination because of multidrug-resistant Acinetobacter baumannii surrounding colonized or infected patients. Am J Infect Control.

[41] Amankwah S, Adisu M, Gorems K (2022). Assessment of phage-mediated inhibition and removal of Multidrug-Resistant Pseudomonas aeruginosa Biofilm on Medical implants. Infect Drug Resist.

[42] Belayneh Getachew AT, Tsehaye Asmelash H, Taddle, Araya G. Therapeutic potential of bacteriophage isolated from sewage for multidrug resistant Escherichia coli infection in mice. pmac2018.

[43] Nureye DY, Assefa S, Mohammed S (2018). Bacteriophages and their applications: general aspects and a new insight in Ethiopia. J Drug Delivery Ther.

[44] Adugna Leta M, Yohannes KT (2017). Assessment of Therapeutic potential of bacteriophages to Control Escherichia Coli Infection in Swiss mice. Model EthiopJApplSci Technol.

[45] Cha K, Oh HK, Jang JY (2018). Characterization of two Novel bacteriophages infecting Multidrug-Resistant (MDR) Acinetobacter baumannii and evaluation of their therapeutic efficacy in vivo. Front Microbiol.

[46] Jeon J, Park JH, Yong D (2019). Efficacy of bacteriophage treatment against carbapenem-resistant Acinetobacter baumannii in Galleria mellonella larvae and a mouse model of acute Pneumonia. BMC Microbiol.

[47] Hasan Ghajavand BN, Esfahani A, Havaei (2017). Isolation of bacteriophages against multidrug resistant Acinetobacter baumannii. Res Pharm Sci.

