# Correction: Isolation of virulent phages against multidrug-resistant acinetobacter baumannii recovered from inanimate objects of Jimma Medical Center, Southwest Ethiopia

**DOI:** 10.1186/s12879-023-08915-4

**Published:** 2023-12-20

**Authors:** Terefe Hailemichael, Lencho Girma, Paulos Fissiha, Alene Geteneh, Tesfaye Kassa

**Affiliations:** 1 Department of Medical Laboratory Science, Mizan Aman College of Health Sciences, Aman, Ethiopia; 2 Department of Medical Laboratory Science, College of Health Sciences, Bonga University, Bonga, Ethiopia; 3 Amhara Public Health Institute (APHI), Bahir Dar, Ethiopia; 4 Department of Medical Laboratory Science, College of Health Sciences, Woldia University, Woldia, Ethiopia; 5 School of Medical Laboratory Science, Jimma University, Jimma, Ethiopia

BMC Infectious Diseases (2023) 23:820

10.1186/s12879-023-08823-7

The original publication of this article contained 2 incorrect table citations. The incorrect and correct information is listed in this correction article. The original article has been updated.

Incorrect.

However, they were sensitive to Doxycycline (100%) followed by Amikacin (95%) (Table 2). Of the 20 isolates recovered, 85% (n = 17) of them were biofilm producers, and recovery of biofilm-forming and MDR A. baumannii was shown to vary with admission wards (Table 3).

Correct.

However, they were sensitive to Doxycycline (100%) followed by Amikacin (95%) (Table 3). Of the 20 isolates recovered, 85% (n = 17) of them were biofilm producers, and recovery of biofilm-forming and MDR A. baumannii was shown to vary with admission wards (Table 2).

