# Supplementary material for: Isolation of virulent phages against multidrug-resistant Acinetobacter baumannii recovered from inanimate objects of Jimma Medical Center, Southwest Ethiopia
Source: BMC Infect Dis. 2023 Nov 22;23:820. doi: 10.1186/s12879-023-08823-7 (PMC10666304; doi:10.1186/s12879-023-08823-7)
Supplement: Supplementary file 1 — Supplementary Material 1 [file 12879_2023_8823_MOESM1_ESM.docx]

Supplement Table 1: OD value of MDR *A. baumannii* isolates with the number of antibiotics resisted at JMC, June-November, 2019

| **Isolate code** | **OD value of biofilm** | **No of antibiotics** |
| --- | --- | --- |
| AB01 | 0.0000 | 3 |
| AB 02 | 0.0000 | 4 |
| AB 08 | 0.0050 | 5 |
| AB 10 | 0.0114 | 7 |
| AB 11 | 0.0130 | 7 |
| AB 12 | 0.0263 | 6 |
| AB 13 | 0.0302 | 5 |
| AB 14 | 0.0369 | 7 |
| AB 15 | 0.0465 | 6 |
| AB 17 | 0.0622 | 8 |
| AB 18 | 0.0940 | 8 |
| AB 20 | 0.1103 | 6 |
